# Treatment of Elsberg Syndrome Causes Fever of Unknown Origin Attributable to Drug Reaction

**DOI:** 10.7759/cureus.18510

**Published:** 2021-10-05

**Authors:** Yash V Bhagat, Elvina Yunasan, Yazan Alzedaneen, Swathi Muttana, Miriam B Michael

**Affiliations:** 1 Department of Medicine, University of Maryland Midtown Campus, Baltimore, USA; 2 College of Medicine, American University of Antigua, Saint Johns, ATG; 3 Internal Medicine, American University of Antigua, New York, USA; 4 Internal Medicine, Howard University, Washington DC, USA

**Keywords:** fever of unknown origin, acyclovir fever, elsberg syndrome, varicella myelitis, hiv aids

## Abstract

A 56-year-old male with a history of non-adherence to HIV anti-retroviral therapy (ART) presented with Elsberg syndrome - varicella reactivation causing fever, painful dermatomal rash, weakness of bilateral lower extremities, and urinary and bowel dysfunction. On the third day of hospitalization, the patient developed altered mental status. An investigation for encephalitis and myelitis revealed a CD4 count of 150 cells/uL, viral load of about 150,000 copies/mL, and MRI of the lumbar spine demonstrating thickening of the cauda equina. Cerebrospinal fluid (CSF) from lumbar puncture confirmed the presence of varicella-zoster virus (VZV). Treatment with acyclovir for 21 days was initiated. However, the patient developed a persistent fever. Evaluation for the source of the fever resulted in identification of anti-viral therapy as the cause. In conclusion, the present report provides a unique example of acyclovir-induced fever developed on treatment of Elsberg syndrome.

## Introduction

Transverse myelitis (TM) is a disorder of the spinal cord that presents with focal inflammation and demyelination, resulting in muscle weakness, sensory deficits, and bowel or bladder dysfunction [1]. It can be caused by various autoimmune, vascular, paraneoplastic, metabolic, or infectious etiologies [2]. Elsberg syndrome is TM resulting specifically from varicella-zoster virus (VZV) or herpes simplex virus-2 infection [3]. VZV is an alpha-herpesvirus with an enveloped double-stranded DNA genome that causes primary herpes zoster infection. Once infected, the virus achieves latency in ganglia along the neuraxis (cranial, dorsal root, and autonomic ganglia), and reactivates if VZV-specific cell-mediated immunity declines, such as in the setting of HIV infection. The reactivated infection may have a complicated disease course, such as the development of post-herpetic neuralgia, meningitis, encephalitis, vasculitis, myelitis, cerebellitis, or retinal necrosis [4]. VZV escape into the cerebrospinal fluid (CSF) often presents with reactive CSF pleocytosis, elevated CSF protein levels, and positive VZV DNA polymerase chain reaction (PCR) results [5]. VZV in the central nervous system is treated with acyclovir intravenously at 10 mg/kg three times a day [6].

Patients with TM may present with a fever of unknown origin (FUO) that may be the result of an independent cause. In an HIV-infected individual, FUO is defined as a temperate of greater than 38.3 C (101 F) for more than three days in-patient or four weeks out-patient [7]. Common etiologies of FUO include infections, malignancies, autoimmune conditions, and other miscellaneous causes, including drug-induced fevers - in this case, due to acyclovir. Although a diagnosis of exclusion, drug-induced fevers may occur with severe spiking fever and relative bradycardia, wherein the heart rate does not rise relative to the rise in fever [8]. Other clues suggesting a drug fever may be rashes, neurological symptoms, and remittance on cessation of drug use.

## Case presentation

**Figure 1:**
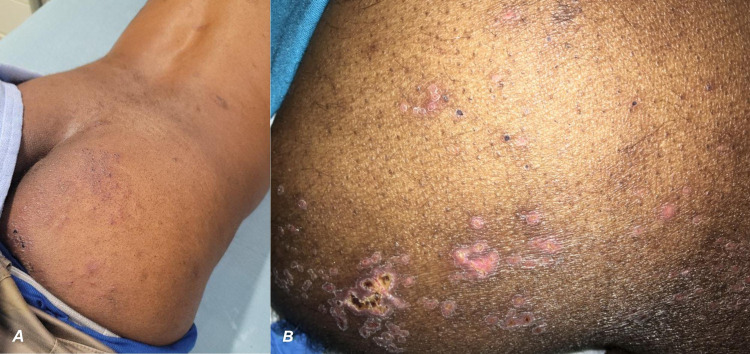
Clinical pictures of herpetic rash A) Localized pruritic vesicopustular rash with scabbing due to varicella zoster virus (VZV) reactivation presenting in the S1-S2 dermatomal distribution on day 1. B) A closer look at the rash on the patient’s buttocks shows scabbing and open sores.

A 56-year-old male patient, first diagnosed with HIV-1 in 1991, had maintained good control of his viral load with compliance to anti-retroviral therapy (ART). However, due to a recent relapse of his alcohol use disorder secondary to his socioeconomic situation during the COVID-19 pandemic, he had been non-adherent with his medications for the past eight months prior to hospitalization. His last known CD4 count from seven months ago was 500 cells/uL with a viral load of 114 copies/mL. On the day of admission, the patient presented with a fever of 102.4 F (39.1 C), difficulty in walking due to lower extremity weakness, burning pain, fecal incontinence, and a rash. The rash and incontinence had developed four days prior to admission while the fever, pain and weakness of the leg had begun only a day prior. The rash was painful, pruritic, and vesiculopustular with some areas of resolution with scabbing (Figure 1A, 1B). It was localized only to his right dorsal S1-2 dermatome, with no midline crossing. Apart from his history of HIV, he has a history of a previous cerebrovascular event treated with lifelong clopidogrel and atorvastatin, hypertension controlled by amlodipine, polysubstance use disorder, and VZV that was once treated with acyclovir.

**Figure 2:**
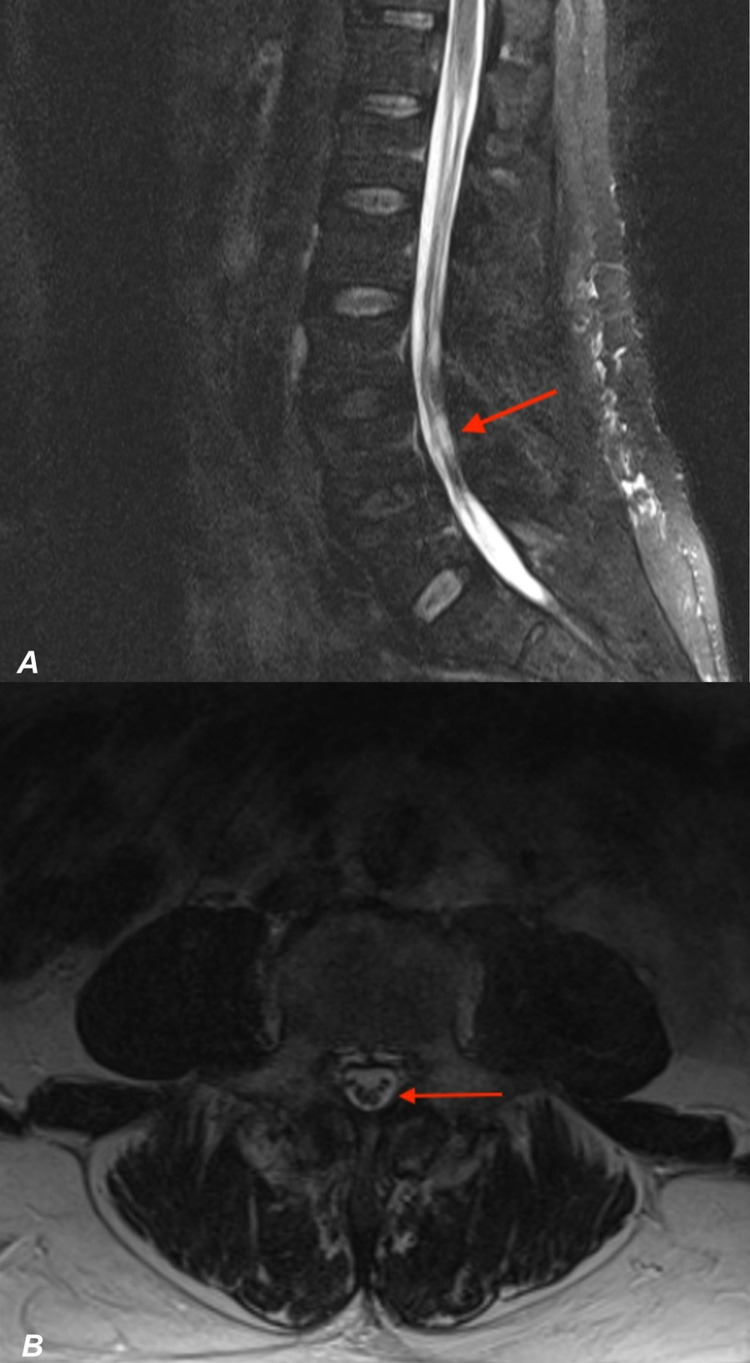
MRI of the spine shows inflammation of cauda equina Longitudinal (2A) and transverse (2B) T2 weighted MRI without contrast of the patient’s lumbar spine demonstrating thickening of the nerve roots of the cauda equina (arrows) and heterogenous appearance of cerebrospinal fluid (CSF) within lower lumbar spinal canal with minimal degenerative anterolisthesis at L4-L5.

On examination, the motor strengths of his bilateral lower extremities were 0/5. Laboratory testing demonstrated reactive HIV antigen and HIV 1 antibody enzyme-linked immunosorbent assays (ELISAs) tests. Furthermore, the HIV viral load was 151,583 copies/mL and the absolute CD4 count was 150 cells/uL. Complete blood count on arrival showed leukocytosis of 11.8 K/mcL (ref 4.5-11) with elevated lymphocyte (30.7%) and eosinophil (6%) count. The patient’s C-reactive protein was also noted to be high (2.8 mg/dl, ref 0-0.99). On day three of hospitalization, the patient, who was still febrile, became agitated with altered mental status. MR imaging of the head was negative; however, MRI of the lumbar spine found heterogeneous CSF and thickening of the cauda equina potentially secondary to infectious cause, along with minimal degenerative disease at L4-5 (Figure 2A, 2B). A regimen of antibiotics for empiric meningitis and encephalitis were started: IV acyclovir 750 mg (10 mg/kg) three times a day, ampicillin 2 g six times a day, ceftriaxone 2 g two times a day, doxycycline 100 mg two times a day, dexamethasone 11 mg four times a day, vancomycin 1.75 g once a day, and Biktarvy (tenofovir-emtricitabine-tenofovir alafenamide) 50-200-25 mg once a day.

Next, lumbar puncture was performed. The CSF was cloudy and xanthochromic in color, with glucose 122 mg/dL (ref 40-70), protein 147 mg/dL (ref 12-60), red blood cells 1110 cells/mcL (ref 0-0), and white blood cells 519 cells/mcL (ref 0-5). The differential of the white blood cells demonstrated a predominance of lymphocytes at 92%. Cytologic reports of the CSF revealed numerous lymphocyte-rich mixed inflammatory cells with mild atypia, suggestive of reactive pleocytosis. We also found that the CSF had no monoclonal B cells. CSF cultures did not grow bacteria, fungi, or acid fast bacteria. Laboratory testing of blood, CSF, or urine was negative for antibodies to toxoplasma, Lyme disease (*B. burgdorferi*), West Nile virus, and cytomegalovirus, PCR for Epstein-Barr virus and herpes simplex virus DNA, RPR for syphilis, and antigens for *Histoplasma* and *Cryptococcus*. However, VZV DNA was positively identified in the CSF. CSF was also sent for PCR testing of a panel of other microbes: *E. coli*, *H. influenzae*, *L. monocytogenes, N. meningitidis, S. agalactiae, S. pneumoniae*, cytomegalovirus, enterovirus, herpes simplex virus 1 and 2, human herpesvirus 6, human parechovirus, VZV, and *Cryptococcus neoformans/gattii.* All PCR panel results were negative, except for that of VZV. Additionally, *Cryptosporidium* antigen tests of the blood and the stool were negative. Ova and parasite testing of the stool, microspridium stool tests, and stool culture were negative as well. Thereafter, all medications were discontinued except Biktarvy (tenofovir-emtricitabine-tenofovir alafenamide). A 21-day course of IV acyclovir 750 mg (10 mg/kg) three times a day was also begun.

On day six of admission, the altered mental status, fevers, and relative eosinophilia had resolved, yet the patient noted urinary hesitancy with overflow incontinence. Digital rectal examination identified no prostate enlargement. A bladder ultrasound found a post-void residual of 433 mL. Although the patient had initially complained of lack of sphincter control leading to fecal incontinence, by day 10, his symptoms had changed into that of straining indicative of a neurogenic bladder. Retesting of CD4 count (201 cells/uL) on day 14 showed improvement in the patient’s immune competence; however, the patient developed daily fevers of 102 F (38.9 C) that resolved with acetaminophen. As a result, work-up for fever of unknown origin (FUO) was begun.

A fever spike curve was obtained by pausing the use of acetaminophen, but it did not correspond to the administration of medications (Biktarvy or acyclovir). Moreover, he did not have any episodes of bradycardia during these fevers, maintaining a normal heart rate throughout the episodes. The erythrocyte sedimentation rate was 129 mm/hr (ref 0-15) and C-reactive protein was 7 mg/dL (ref 0-0.99). An indium-111 nuclear medicine body scan showed no foci of infection or inflammation. Vasculitis studies for antinuclear antibodies (ANA) IgG, antineutrophil cytoplasmic antibodies (ANCA) IgM and IgG, cardiolipin IgG, IgA, and IgM, beta 2 glycoprotein I IgG, IgA, and IgM antibodies were not revealing. Moreover, C3 and C4, partial thromboplastin time (PTT) and corresponding mixing studies, rheumatoid factor, angiotensin-converting enzyme (ACE), and ferritin levels were also found to be normal, and temporal artery and lower extremity duplex ultrasound showed no stenosis or thrombosis. However, the levels of eosinophils had inexplicably increased back up to 5.9%. After completing 21 days of treatment with acyclovir, the fever resolved with cessation of acyclovir. At that point, the patient’s rash had resolved, muscle strength in bilateral lower extremities progressively improved to 5/5, and fecal and urinary incontinence partially rectified. However, neuropathic pain persisted in the patient’s right dorsal S1-S2 dermatome. The patient was successfully housed at a sober home post-discharge due to his polysubstance use disorder. One week post discharge, the patient noted complete resolution of his neurogenic bowel, but persistence of the neurogenic bladder. At a one-month follow-up at the urologist, the patient mentions having complete resolution of the neurogenic bladder too.

## Discussion

In HIV-compromised individuals, the reduced CD4+ T cell count leads to a decrease in Major Histocompatibility Complex Class II molecules that play a role in the immune response to viruses. This compromised state increases the likelihood of VZV reactivation [9]. While the low CD4 count may be related to the recurrence of VZV, it is not a good indicator of the risk of complications. Myelitis, a rare complication of VZV, generally occurs within three weeks of the herpetic rash [10]. In our patient, the herpetic rash and TM occurred four days apart. Mechanistically, the MRI of our patient confirmed the presence of TM resulting in symptoms like those of the cauda equina syndrome, thus attributing the patient’s motor symptoms and neurogenic bladder and bowel to the involvement of sacral nerve roots and detrusor muscle areflexia [11]. Acyclovir, a nucleoside analog that inhibits the replication of viruses, has been successfully used to treat VZV and herpes simplex virus, including cases with the cauda equina syndrome [12]. Although several mechanisms of resistance to acyclovir have been elucidated in AIDS-compromised individuals [13,14], acyclovir is generally well tolerated with few side effects, including acute kidney injury, hepatotoxicity, and neurotoxicity [15-17]. Interestingly, although cases of rare acyclovir-induced diseases exist [18], there is only one prior case to our knowledge that demonstrates a drug-induced fever with systemic acyclovir use [19]. While in both cases the initial fever due to the viral infection resolved prior to the occurrence of the FUO and the patients in each case had prior treatments with acyclovir in the past which may have been the sensitizing event, the drug fever of the patient in Shea et al. was persistent and was not reduced by acetaminophen as opposed to our case where the fever showed a spiking pattern with normal intermediate temperatures and responded well to the use of acetaminophen. Additionally, neither case developed a new rash, neurological symptoms, myalgia, or gastrointestinal changes that coincided with the occurrence of the FUO. Paralleling what was noted in Shea et al., which found no fever 24 hours after the last dose of acyclovir, our patient's fevers resolved the morning after the last dose of acyclovir was administered.

Interestingly, unlike the lack of eosinophilia seen in the case report by Shea et al., we found that our patient had high absolute eosinophil counts on admission that had gradually resolved prior to increasing again. Drug reaction with eosinophilia and systemic symptoms (DRESS) is another potential differential diagnosis for our patient's symptoms; however, the moderate eosinophilia, lack of rash, lymphadenopathy, and visceral organ involvement make it less likely. Additionally, changes in the level of eosinophilia independent of the application of the inciting medication are unlikely to occur in DRESS. Immune reconstitution inflammatory syndrome (IRIS) was also a differential that was considered due to the presentation of the FUO at two weeks post resumption of ART. Yet, the abrupt pause in fevers on discontinuation of acyclovir while continuing administration of ART deemed IRIS unlikely as well. 

Moreover, acyclovir, famciclovir, and valacyclovir have allergic and systemic cross reactions that are hypothesized to exist due to a shared 2-aminopurine nucleus [20]. As a result, these medications may not be viable options to treat VZV myelitis in individuals with drug reactions. Additionally, the lack of evidence demonstrating the use of alternative medications, such as foscarnet and cidofovir, for VZV myelitis treatment has generated a novel investigative question for future research.

## Conclusions

We present a unique case of an HIV-positive individual who presented with Elsberg syndrome due to non-compliance with his ART medication and was treated with IV acyclovir that caused a drug-induced fever. This case warrants further research into the mechanism underlying acyclovir-induced fevers, methods to avoid it, and exploration of alternative anti-viral treatments for VZV myelitis.
